# Attentional templates for target features versus locations

**DOI:** 10.1038/s41598-024-73656-6

**Published:** 2024-09-27

**Authors:** Mikel Jimenez, Ziyi Wang, Anna Grubert

**Affiliations:** Department of Psychology, University of Durham, Upper Mountjoy, South Rd, Durham, DH1 3LE UK

**Keywords:** Visual attention, Attentional templates, Spatial attention, Feature-based attention, ERP, N2pc, Attention, Human behaviour

## Abstract

Visual search is guided by visual working memory representations (i.e., attentional templates) that are activated prior to search and contain target-defining features (e.g., color). In the present study, we tested whether attentional templates can also contain spatial target properties (knowing where to look for) and whether attentional selection guided by such feature-specific templates is equally efficient than selection that is based on feature-specific templates (knowing what to look for). In every trial, search displays were either preceded by semantic color or location cues, indicating the upcoming target color or location, respectively. Qualitative differences between feature- and location-based template guidance were substantiated in terms of selection efficiency in low-load (one target color/location) versus high-load trials (two target colors/locations). Behavioral and electrophysiological (N2pc) measures of target selection speed and accuracy were combined for converging evidence. In line with previous studies, we found that color search was highly efficient, even under high-low conditions, when multiple attentional templates were activated to guide attentional selection in a spatially global fashion. Importantly, results in the location task almost perfectly mirrored the findings of the color task, suggesting that multiple templates for different target locations were activated concurrently when two possible target locations were task relevant. Our findings align with accounts that assume a common neuronal network during preparation for location and color search, but regard spatial and feature-based selection mechanisms as independent.

## Introduction

Visual search is guided by target representations that are held in visual working memory, i.e., attentional templates^1,2^. Attentional templates contain target-defining features, such as color, shape, or size, and are activated in preparation for search^3,4^. Any object that matches the content of the template will consequently be selected with priority^5–9^. Because the target location in visual search is typically unknown, it is assumed that feature-based attentional templates guide attention in a spatially global fashion across the visual field^10–13^.

Empirical evidence supporting this assumption comes, for example, from Berggren et al.^14^. Their search displays contained two targets that were defined by a specific color/location configuration (e.g., red in the upper and blue in the lower visual field). Search displays were preceded by spatially uninformative color cues that either matched this color/location arrangement (red over blue), appeared in the reverse color/location arrangement (blue over red), or had task-irrelevant colors (pink over orange). Berggren et al.^14^ recorded EEG and measured the N2pc component of the event-related potential (ERP), as an electrophysiological marker of attentional selection, in response to these cues. The N2pc is an increased negativity, elicited at around 200 ms after stimulus onset at posterior electrode sites over extrastriate visual cortex^15^, contralateral to the side of an attended object^16–18^. All cues that contained the target colors, irrespective of whether they were shown in the exact color/location arrangement, or the reversed arrangement, triggered comparable N2pc components, demonstrating that these cues were attended. Task-irrelevant color cues were ignored and never triggered any N2pcs. This demonstrates that color-specific attentional templates selectively guide attention to all color-matching objects across the whole visual field in parallel.

In contrast to such feature-based spatially global guidance, spatial cueing tasks have demonstrated that attention can efficiently be guided to specific target locations without any prior knowledge about the featural target identity^19,20^. But does that mean that attentional templates can contain spatial rather than featural target information? And if so, is target selection guided by such location-specific templates more or less efficient than selection guided by feature-specific templates that operate in a spatially global fashion across the visual field? These questions are by no means arbitrary because feature- and location-based information are processed in two separate cortical processing streams^21–23^. Stimulus features, such as color or shape, are thought to be processed in occipitotemporal visual areas (ventral stream), and location-based information, including motion, are thought to be processed in occipitoparietal visual areas (dorsal stream).

Studies across a wide range of paradigms and measures have since mapped location-based and feature-based attentional mechanisms onto these two processing streams^24–26^ (but see^27^, for a comprehensive review of evidence supporting a not so strict separation of the two pathways) and have examined efficiency differences between location- and feature-based selection. With this respect, the predominant view of the field is that spatial selection exceeds feature-based selection. For example, spatial cueing effects (faster reaction times for targets at spatially cued as compared to uncued locations) were found to be increased when the target location as compared to the target color was task relevant^28^, or even absent when the target shape was relevant^29^. Kwak and Egeth^30^ observed inhibition of return only for target location repetitions but not for target color or orientation repetitions. Tracking the time course of spatial versus feature-based cueing effects, it was reported that spatial cueing effects (measured in target detection accuracy d’) improved with short cue-target intervals (~ 300 ms), while feature-based cueing effects took more than 500 ms to establish^31^. Furthermore, neuroscientific evidence showed that early ERPs after target onset (P1, N1 components) were increased for targets at cued locations, irrespective of whether that target was shown in a cued or uncued color^32^. Finally, baseline shifts measured in fMRI were dominated by the expected location of an upcoming target rather than the cued featural identity (motion or color) of that target^33^.

However, the caveat with spatial cueing studies like the ones reported above is the spatial cue per se because it provides a salient bottom-up signal that directs spatial attention to the target location. This is especially true for exogenous location cues that directly indicate the upcoming target location^28^. But even central arrow cues^29,31,34^ or task instructions about the relevant target side^32,33^, might prevent participants from setting up an actual attentional template. Why would they need to activate a mental representation of a target location in visual working memory, when attention can simply be guided by a spatial salience signal?

In the present study, we aimed to address this caveat by comparing feature-based (knowing what to look for) versus location-based (knowing where to look for) attentional guidance in visual search in which target colors versus locations were cued by means of semantic (rather than perceptual) cues, i.e., the first letter of a color word versus a numbered location in the search display. With semantic cues, observers must form a memory representation of the target color or location and activate a color or location template, respectively, to be able to identify the target among the distractors. To ensure that memory representations were held active in working memory rather than being handed off to a cognitively less demanding long-term storage, a different target color or location was cued in every trial^35,36^. We measured behavioral and electrophysiological indices of target selection speed (reaction times and N2pc onset latencies) and accuracy (error rates and N2pc amplitudes) in color versus location search. All search displays contained four differently colored horizontal and vertical bars that were randomly located at four out of eight possible stimulus locations. Participants’ task was to find the bar in the cued color, or at the cued location, and report its orientation. That meant that physical search displays and task demands were identical in the two tasks and ERPs were not contaminated with perceptual or response-related asymmetries (Hillyard Principle^37^). If location-based selection is superior to feature-based selection, N2pc components should be earlier and larger in the location than the color task, and this should also be reflected in faster reaction times (RTs) and lower error rates in location than color search.

Qualitative differences of attentional guidance by feature-based and spatial templates were substantiated in terms of selection efficiency under low versus high memory load conditions. In low-load trials, a single target color/location was cued, in high-load trials, participants were presented with two color/location cues simultaneously. This manipulation enabled us to assess selection efficiency as a difference variable, in terms of load costs (e.g., high-load minus low-load RTs), which would not be confounded by any potential main effect of task difficulty. In a previous study from our lab^38^, participants had to identify two color-defined targets that were presented in rapid succession. The two targets were either shown in the same or in two different colors. RTs in the two-color condition were considerably slower than in the one-color condition (+ 139 ms). While these pronounced RT load costs evidently suggest a substantial decrease in selection efficiency when two color templates had to be activated for search, the N2pc results looked very different. The two successive targets triggered reliable N2pc components that overlapped in time, indicating that the two targets were selected in parallel both when they had the same or two different colors. The N2pcs triggered in the two-color condition were attenuated and delayed, but the amplitude differences were statistically not reliable, and the N2pc onset delays were numerically significantly smaller (+ 20 ms on average) than the behavioral load costs. These N2pc results clearly show that two color templates can be activated concurrently and guide attention to multiple targets with different colors in parallel. The N2pc latency delays associated with the increased template load were caused by mutually competitive interactions between the two simultaneously activated attentional templates (see^39^, for similar findings). The increased load effects at the level of the RTs were therefore not related to selection efficiency (as measured by the N2pc) but were instead triggered at later processing stages that follow target selection (e.g., target identification^9^).

We expect to replicate these previous patterns of behavioral and electrophysiological load costs in the color task of this experiment. RTs should be substantially delayed in high-load as compared to low-load trials. N2pc components should also be delayed and attenuated in trials in which two colors, rather than one, are task relevant, but these load costs should be insignificant in comparison to the RT load costs. The question is whether there will be a similar pattern of load effects in the location task, and whether selection efficiency will be comparable in the two tasks. Load effects should be comparable in the two tasks if location-specific templates are as efficient in guiding attention to multiple target locations as feature-based templates. However, if spatial selection is more efficient that feature-based selection [e.g., ^28–33^, load effects should be significantly smaller in the location than the colour task, especially at the behavioural level. Alternatively, it might also be possible that spatial templates are less efficient under high-load conditions than color templates because they are spatially specific rather than global [e.g.,^10–12^. In that case, load effects should be substantially increased as compared to the color task, especially at the level of the N2pc. To provide additional statistical evidence for any potential null results, all standard parametric statistical tests were complemented with Bayesian statistics.

## Methods

### Participants

Nineteen Durham University undergraduate and graduate students were tested in this Experiment. The experiment was approved by the Ethics Committee of the Psychology Department at Durham University and was conducted in accordance with the Declaration of Helsinki. Participants gave informed written consent prior to testing. Five participants were excluded from analysis due to excessive eye movement activity, which led to a loss of > 30% of the trials in at least one of the four task conditions during artefact rejection. Nine of the remaining participants were female, five were male. They were aged between 19 and 42 years (*M* = 24.4, *SD* = 7.2) and two were left-handed (the other 12 were right-handed). All participants had normal or corrected-to-normal visual acuity and normal color vision (tested with the Ishihara color vision test^40^). They either received course credits or were paid (£10 per hour) as a compensation for their time. The sample size of 14 was calculated by means of an a priori power analysis using MorePower 6.0^41^ to detect an interaction in a 2 × 2 × 2 factorial repeated measures ANOVA (within-subjects) with an assumed alpha of 0.05 and power of 0.80, and a moderate effect size (η^2^_p_) of 0.4.

### Stimuli and procedure

**Fig. 1:**
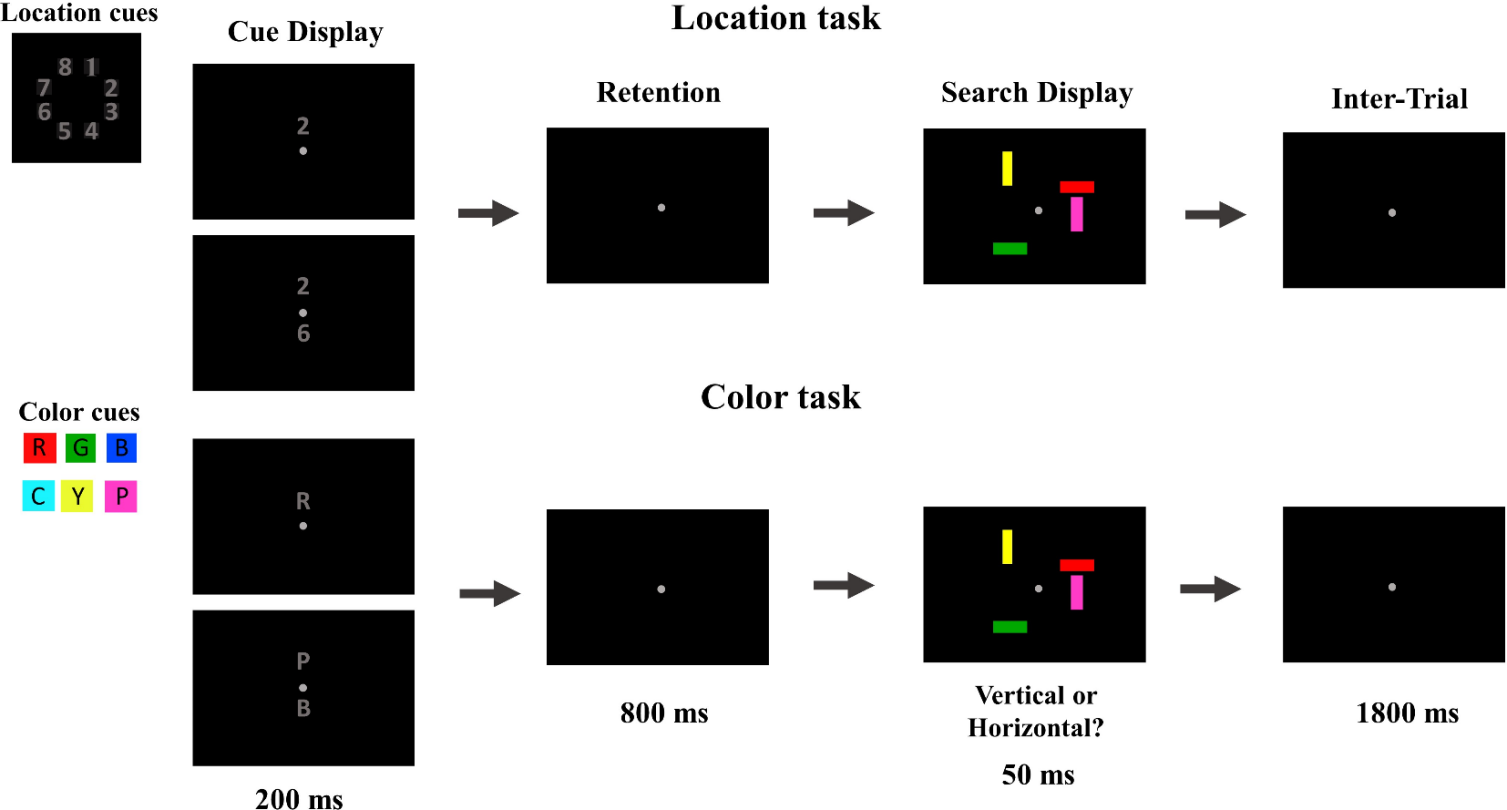
Schematic illustration of the time course and the stimuli presented in each trial of the location (top panel) and color task (bottom panel).

Participants were seated in a sound attenuated, dimly lit Faraday cage. Stimuli were presented on a 24-in BenQ LCD monitor (resolution: 1280 × 1024 pixels; 75 Hz refresh rate) at a viewing distance of approximately 100 cm. Stimulus presentation, timing and response collection was controlled by a LG Pentium PC running under Windows 7, using MATLAB and the Cogent 2000 toolbox. All stimuli were presented on a black background. A central grey fixation point (CIE x/y color coordinates: 0.313/0.352; 0.3° × 0.3° of visual angle) was continuously shown throughout each experimental block. Each trial began with the presentation of a cue display (200 ms), which indicated the target color(s) or location(s) in each trial. The cue display was followed by an 800 ms retention period, the response-relevant search display (50 ms), and a 1800 ms inter-trial interval during which responses were collected (Fig. 1).

Search displays contained four vertically or horizontally oriented bars (0.6° × 1.15° for each bar), which were located on an imaginary circle (1.3° eccentricity from fixation with respect to the center of the bars) with eight possible stimulus locations (four in each hemifield). All stimulus locations were equidistant. Always two bars were presented in each hemifield. Each bar was shown in a different color. There were eight possible colors: red (CIE x/y color coordinates: 0.629/0.344), green (0.283/0.613), blue (0.175/0.202), pink (0.306/0.194), yellow (0.461/0.478), cyan (0.214/0.340), grey (0.313/0.342), and brown (0.422/0.462). All colors were equiluminant (∼11.0 cd/m^2^). Participants’ task was to report the orientation (vertical/horizontal) of a pre-cued target bar by pressing the arrow up/down key on a standard keyboard as quickly but accurately as possible. A new target identity, either the target color or the target location, was defined in each trial by the cues that preceded the search display.

Cue displays contained either one (low-load, 50%) or two (high-load, 50%) grey alphanumerical objects (0.6° × 0.6°) that were presented above, or above and below the fixation point, respectively (0.4° eccentricity from fixation with respect to the object center). In the *Color Task*, cues were capital letters that represented the initial letters of the upcoming target color (R = red, G = green, B = blue, Y = yellow, P = pink, and C = cyan; see Fig. 1). Note that grey and brown were only ever used as nontarget colors in the search display and were never cued as target colors. Each of the six possible target colors in the low-load condition and each of the 15 possible target color combinations in the high-load condition were cued equally often. In the high-load condition, the location (above/below of fixation) of the two letters was fully randomized. Only one of the two cued target colors appeared in the search display. In the *Location Task*, cues were digits that represented the upcoming target location. The eight possible target locations on the imaginary circle were numbered clockwise starting with the top position in the right hemifield (1 = 22.5° to the right from the vertical midline, 2 = 67.5°, 3 = 112.5°, 4 = 157.5°, 5 = 202.5°, 6 = 247.5°, 7 = 292.5°, and 8 = 337.5°; see Fig. 1). Each of the eight possible target locations in the low-load condition and each of the 28 possible target location combinations in the high-load condition were cued equally often. The positions of the two digits in the high-load condition (above/below of fixation) were randomly drawn in each trial. In high-load trials only one of the two cued target locations was occupied by a search item (the target), the other cued location was empty in the search display.

 The search displays in the two task conditions were physically identical to ensure the comparability of the N2pc components measured in response to the target bars in these two tasks. In both tasks, the target bars were presented equiprobably at any of the eight possible stimulus locations and were equally often shown in any of the six possible target colors, even though the target locations were response irrelevant in the color task and the target colors were response irrelevant in the location task. Nontarget colors and locations were randomly determined in each trial from the sets of un-cued colors and locations, respectively. The response-to-key mapping (vertical/horizontal response on arrow up/down key) and the hand-to-key mapping (left/right hand on arrow up/down key) were counterbalanced across participants but were kept constant for each participant for the duration of the whole experiment. The color and location tasks were tested in different blocks and the order of task was counterbalanced across participants. Within each block, template load (one/two target color/locations) and target side (left/right hemifield) combinations appeared equally often (12 trials for each combination), resulting in a total of 48 trials per block. Twelve blocks were tested for a total of 576 trials per task (~ 40 min run time per task). Before each task, participants received a few practice trials until they felt comfortable with the task (~ 24 trials). These practice data were not recorded.

### EEG recording and data analyses

EEG was DC-recorded from 25 scalp sites (at standard positions of the extended 10/20 system; EasyCap, Brain Products), sampled at 500 Hz (BrainAmp DC amplifier, Brain Products), and digitally low-pass filtered at 40 Hz (no other filters were applied after data acquisition). Impedances were kept below 5kΩ. Data was online referenced to the left earlobe and re-referenced offline to the average of both earlobes. EEG was segmented into 500 ms time windows, including a 100 ms pre-stimulus baseline and a 400 ms ERP time window after search display onset. Trials with anticipatory (< 200 ms) or very slow responses (> 1500 ms), together with missing or incorrect responses were excluded from the analysis. In addition, data contaminated with artifacts (blinks exceeding ± 60 μV at FPz; eye movements exceeding ± 30 μV in the bipolar horizontal EOG channel; muscular movements exceeding ± 80 μV in all other channels) were also excluded from EEG analyses. Artifact rejection resulted in an exclusion of 9.73% (*SD* = 6.82%; ranging between 2.30% and 26.97% across participants) and 8.89% (SD = 6.70%; ranging between 2.86% and 29.19% across participants) of all segments in the color and location task, respectively. The remaining segments were averaged separately for each task (color versus location) and template load (low- versus high-load) in which the search targets were in the left versus right hemifield. N2pc components were quantified based on ERP mean amplitudes obtained in the 220–320 ms time window after target onset at lateral posterior electrodes PO7 and PO8, contralateral and ipsilateral to the side of the target. N2pc onset latencies were determined with a jackknife-based procedure^42^. Separately for each task and template load condition, 14 subsample grand-average difference waves (contralateral minus ipsilateral ERPs at PO7/8) were computed, each excluding one different participant from the original sample. N2pc onset latencies were defined as the point in time when each subsample difference wave reached an absolute onset criterion of − 0.5 µV (see^7^, for identical procedures), and were then submitted to a repeated measures ANOVA. To the best of our knowledge, the issue of how to perform non-parametric statistical tests on jack-knifed data (i.e., to decrease artificially inflated Bayes factors due to reduced error variance in jack-knifed data), has not yet been discussed in the literature. No Bayesian statistics were therefore performed for N2pc latency analyses. The statistical values of the *F* test comparing N2pc onset latencies were power corrected according to the formula described by Ulrich and Miller^42^, as indicated with the label *F*_c_. Generally, all *t*-tests reported are two-tailed and Bonferroni corrected were necessary. Effect sizes are reported in terms of Cohen’s *d*^43^, with a confidence interval of 95%, for *t*-tests, and partial eta squared (η^2^_p_) for *F*-tests (labelled η^2^_pc_ for power corrected jack-knifed data). EEG data processing was conducted with the BrainVision Analyzer software (Brain Products GmbH, Gilching, Germany). All statistical analyses were performed using RStudio (Version 2022–02-03–492, RStudio, 2022, based on the R programming language Version 4.2.0, R Core Team, 2022) using the companion to applied regression (CAR^44^) package for null hypothesis significance testing (NHST) analyses and JASP statistical software (version 0.16.4, www.jasp-stats.org) for Bayesian tests (see^45^ for a methodological justification on running Bayesian ANOVAs on JASP, see^46^ for similar analyses). As prior distribution on the fixed effects, we specified the default multivariate Cauchy distribution (scale of -sqrt(2)/2, sqrt(2)/2), which reflects a lack of information about the parameters^47^. Bayesian Model Averaging^48,49^ was computed across matched models, which compares models that contain a specific effect to equivalent models without the effect, or in other words, it averages the conclusions from each candidate model weighted by that model’s posterior plausibility.

## Results

### Behavioral results

**Fig. 2:**
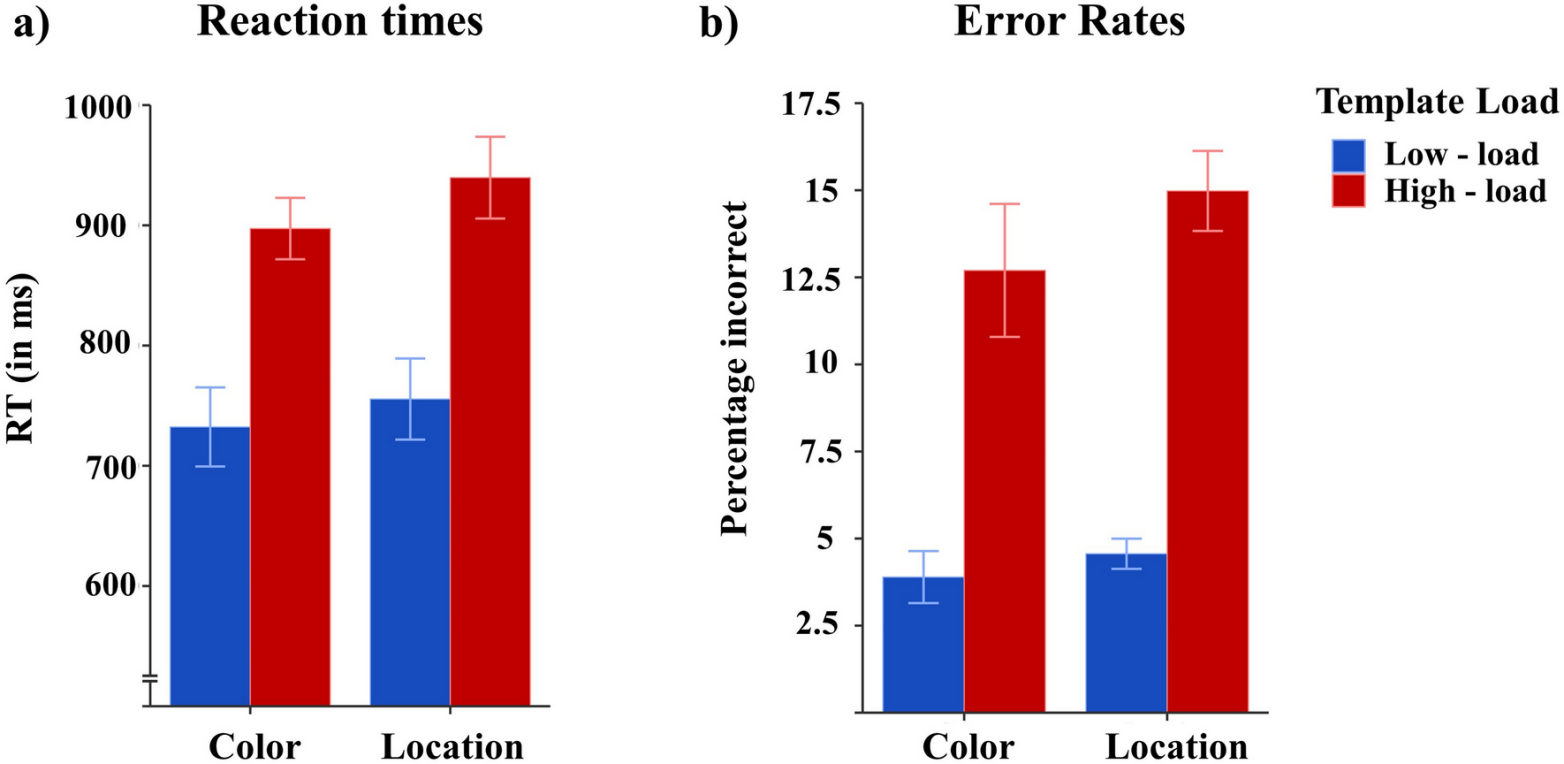
(**a**) Mean reaction times (in milliseconds; left panel) and (**b**) error rates (percentage incorrect trials; right panel) in low-load (blue bars) and high-load trials (red bars) of the color and location tasks. Error bars represent mean standard errors.

Anticipatory (< 200 ms) and very slow responses (> 1500 ms) were removed from analysis (2.0% and 4.5% of all trials in the color and location task, respectively). Figure 2 shows mean correct reaction times (RTs; left panel) and error rates (right panel) in the low- and high-load conditions of the color and location tasks. RTs and error rates were sent to two separate repeated measures ANOVA with the factors Task (color vs location) and Template Load (low vs high). Both ANOVAs on RTs and error rates revealed main effects of Template Load, both *F*(1,13) > 119.8, *p* < 0.001, η^2^_p_ > 0.90, because RTs were slower and error rates were higher in high- (918 ms, 13.8%) than low-load trials (744 ms, 4.2%) when two as compared to one target identity was cued. However, none of the effects involving the factor Task reached significance, all F(1,13) < 3.5, p > 0.082, η^2^_p_ < 0.21, which suggests that the template load effects on RTs and error rates were comparable in both the color (165 ms, 8.9%) and location task (184 ms, 10.4%). Two Bayesian ANOVAs supported these conclusions. For both the RT and error rates data, Bayesian Model Averaging provided clear evidence in favor of the Template Load effect (RTs: BF_incl_ > 100; Error rates: BF_incl_ > 100), but not for an effect of Task (RTs: BF_incl_ = 1.378; Error rates: BF_incl_ = 0.642) or a Task x Load interaction (RTs: BF_incl_ = 0.668; Error rates: BF_incl_ = 0.536).

### N2pc components

**Fig. 3:**
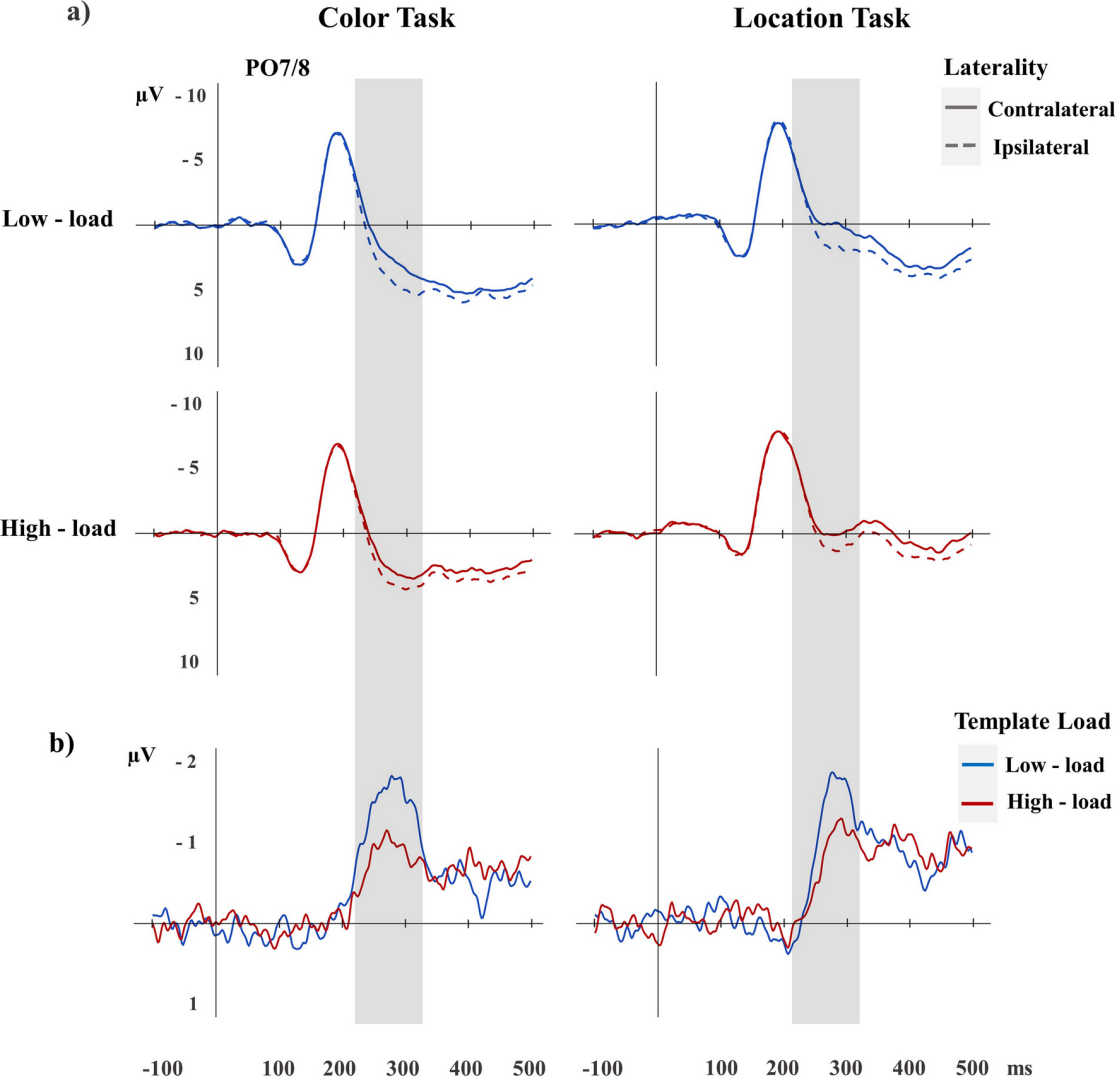
(**a**) Grand-averaged ERPs (in microvolts; top panels) elicited in the search displays at electrode sites PO7/8 contralateral and ipsilateral to the color (left panels) or location targets (right panels) in low-load (blue lines) and high-load trials (red lines), (**b**) together with the corresponding contralateral-ipsilateral N2pc difference waveforms. Shaded areas indicate N2pc time windows (220–320 ms after search display onset).

Figure 3 (top panels) shows grand-averaged event-related potentials (ERPs) elicited at electrode sites PO7 and PO8 contra- and ipsilateral to targets in low- and high-load trials of the color (left panels) and location tasks (right panels). N2pc components, substantiated as increased negativity in contralateral as compared to ipsilateral ERPs in the 220–320 ms time window post search display onset, were triggered in all four conditions. However, these N2pcs seemed to be attenuated in high- relative to low-load trials, when two as compared to one target definition was cued. These N2pc differences can be seen more clearly from the difference waveforms in Fig. 3 (bottom panel), obtained by subtracting ipsilateral from contralateral ERPs, separately for low and high template load trials in the color (left panel) and location tasks (right panel).

To verify these observations statistically, N2pc mean amplitudes were subjected to a repeated measures ANOVA with the factors Task (color vs location), Template Load (low- vs high-load), and Laterality (electrode contralateral vs ipsilateral to target). There was a main effect of Laterality, *F*(1,13) = 51.03, *p* < 0.001, η^2^_p_ = 0.80, and a significant interaction between Template Load and Laterality, *F*(1,13) = 14.55, *p* = 0.002, η^2^_p_ = 0.53, demonstrating that N2pcs were attenuated in trials in which participants searched with two as compared to a single target template. However, follow-up *t*-tests confirmed that solid N2pcs were triggered in both low (− 1.30 µV), *t*(1,13) = − 6.45, *p* < 0.001, *d* = − 1.72, and high template load conditions (− 0.8 μV), *t*(1,13) = − 7.46, *p* < 0.001, *d* = -1.99. Furthermore, neither the Task*Laterality, nor the 3-way interaction reached significance, both *F*(1,13) < 1.11, *p* > 0.309, η^2^_p_ < 0.07, which suggests that N2pc components were comparable between the color (− 1.2 µV) and location tasks (− 1.0 µV) and even more importantly, that the template load effects (high- minus low-load N2pc amplitudes) were statistically the same in the color (0.6 µV) and location task (0.5 µV). All these conclusions were supported by a complementary Bayesian ANOVA. Bayesian Model Averaging provided evidence only in favor of the Template Load x Laterality interaction (BF_incl_ of 6.070), but not for a Task x Laterality interaction (BF_incl_ of 1.233). Overall, results from the NHST and Bayesian tests suggest that the data was best explained by the three main interactions together with the Template Load x Laterality interaction effect, showing that N2pc components were equally attenuated in the high-load condition in both tasks.

Onset latencies, measured at − 0.5 µV of the jack-knifed N2pc difference waves, were subjected to a repeated-measures ANOVA with the factors Task and Template Load. N2pcs were delayed in high- (243 ms) as compared to low template load trials (229 ms), *F*_c_(1,13) = 4.97, *p* = 0.018, η^2^_pc_ = 0.28. N2pcs were furthermore delayed in the location (248 ms) as compared to the color task (225 ms), *F*_c_(1,13) = 5.92, *p* = 0.012, η^2^_pc_ = 0.31, mirroring the numerical (but not statistical) differences in overall RTs (see Discussion). However, the Task*Template Load interaction failed to reach significance, *F*_c_(1,13) = 0.06, *p* > 1, η^2^_pc_ < 0.01, demonstrating one more time that the effects of template load were identical in the color (16 ms) and the location task (11 ms).

## Discussion

The goal of this study was to test whether location-specific attentional templates can be employed to guide attention to one or two target locations in the absence of a spatial salience signal, and if so, whether selection would be as efficient when guided by such location-specific versus spatially global color templates. We employed a cueing paradigm in which the upcoming target color or location, respectively, was cued by means of alphanumerical characters. Cueing the target colors and locations semantically, rather than perceptually, avoided any salience-based bottom-up guidance in the search arrays. To ensure that search templates were held active in working memory, a new color or location was cued in every trial. Selection efficiency was substantiated in terms of load effects, i.e., difference measures of performance in high- versus low-template load conditions.

The results in the color task are perfectly in line with previous studies that have demonstrated concurrent activation of multiple color-specific attentional templates in a variety of tasks and measures^50–58^. Selection speed was mainly reduced at the behavioral level (high-load minus low-load RTs; 165 ms), while the N2pc latency costs were much smaller (16 ms). Behavioral load costs like these have previously been interpreted to reflect severe capacity limitations of template-guided search. It was suggested that only one attentional template for one target feature can be active at any moment in time^59^ and that consequently, during search for multiple target features, attentional templates need to be switched serially in a time-consuming fashion^60^. However, this single template account^2^ has since been revised^39^, and it is now accepted that the significant, but very small N2pc latency differences reflect mutually competitive interactions between two simultaneously activated templates rather than serial template switch costs^38,39,61^. The increased RT load costs are thought to be generated at later processing stages, after target selection, when the target must be identified^9^. Load effects also affected selection accuracy. Error rates were increased in high-load as compared to low-load trials, demonstrating that our data were not confounded with a speed-accuracy trade-off. Furthermore, N2pc amplitudes were reduced in two-color as compared to one-color trials, and this amplitude reduction was significant. This might mean that some targets were not selected within the N2pc time window on a substantial number of trials when two colors were task relevant. This result is somewhat surprising because in most of our previous experiments, N2pc amplitudes between high- and low-load conditions were statistically comparable^38,53,54,62^. In our previous studies, however, target colors were typically fixed for each participant, and low-load and high-load trials were blocked. In the present experiment, we intentionally cued the target color(s)/location(s) on a trial-by-trial basis and intermixed high- and low-load trials to make sure target templates were held active in working memory until the target was found. We used a similar trial-by-trial cueing design in one previous study^35^, in which load-dependent N2pc amplitude differences were indeed reliable, too. It is possible that the increased target variability together with the load uncertainty caused by the trial-by-trial cueing increased working memory demands and resulted in more pronounced load effects, at least at the level of selection accuracy as indexed by N2pc amplitudes.

More importantly, and with respect to our original research question, results in the location task almost perfectly mirrored the findings of the color task. Selection speed decreased in high- as compared to low-load trials but again, this effect was much more pronounced at the level of RTs (184 ms) than N2pc onset latencies (11 ms). Load effects also reduced search accuracy, both in terms of behavioral error rates and attenuated N2pc components. Critically, all load effects in the location task were statistically identical to the color task. None of the interactions involving the factor task for any of our measures reached statistical significance and that was also confirmed by additional Bayesian statistics. From this pattern of results, it can be concluded that color and location templates guide target selection equally efficiently.

 Our findings are at odds with the assumption that spatial attention is superior to feature-based selection^21,28–30,32,33^. If anything, in the present experiment processing speed was faster in the color than the location task (numerically for RTs, but significantly for N2pc onset latencies). Measures of selection accuracy (i.e., error rates, N2pc amplitudes) were statistically identically in the color and location task. This lack of main effects of task demonstrates that controlling task difficulty is key when comparing feature- versus location-based selection. In the present experiment, we employed semantic cues (digits/letters) to equate the perceptual input used to activate the search templates for the upcoming trial. Instead, in two pilot experiments (see Supplementary Materials for detailed methods and results), we used exogenous cues (colored squares presented at the actual target locations) and central indicators (colored dots presented next to fixation pointing into the direction of the target locations) and replicated previously observed faster RTs in the location as compared to the color task both with exogenous and central cues (see Supplementary Materials). This suggests that the superiority effect for spatial selection over feature-based selection found in previous studies might be a confound of imbalanced guidance mechanisms. While actual feature representations had to be activated for attentional guidance during feature search, in location search, attention might have been merely guided by a cue-induced location-based salience signal.

In a recent fMRI study, Egner et al.^63^ found that even though featural and location specific information are processed in two different processing streams, there is a shared network of spatial and feature-based information. Search targets were pre-cued with a color/location combination. Critically, the validity of the cues with respect to the upcoming target color and location was independently manipulated (50%, 70%, 90% cue validity for each target dimensions). Search performance increased with increasing cue validity, and cue-related BOLD responses revealed that spatial and color information were represented additively in shared frontal, parietal, and cingulate regions, during preparation for search. However, the absence of any interactions between spatial and feature-based target detection confirmed that both types of attentional guidance contributed independently to target selection. In terms of template-guided search, this suggests that color- and location-specific representations were set up in a shared neuronal network prior to search, but then guided selection independently to the matching colors or locations, respectively. This interpretation does seem to fit the result patterns of the present experiment very well.

In contrast to Egner et al.^63^, other studies showed that feature-based attention is not independent of spatial attention^64,65^. Advocates of such theories could argue that the null effects observed in the present study are caused by integrated effects of feature-based selection at the target’s location, i.e., attention is guided to the location that contains the relevant feature, rather than the actual feature-contrast per se. Vice versa, it might not be possible to attend to a specific location without also attending to the color that is present at that location, even though color might be completely task irrelevant. Is it perhaps impossible to measure independent color and location selection mechanisms to begin with? Andersen et al.^66^ comprehensively addressed this question in an EEG experiment measuring steady-state visual evoked potentials (SSVEP). In their experiment, participants monitored clouds of dots which contained both dots in a cued target color and an uncued distractor color, which rapidly and randomly changed positions. Participants had to indicate short episodes of luminance reduction via button press. Behavioral as well as electrophysiological indices of attentional selection showed selectively enhanced processing when the luminance changes occurred in the cued as compared to uncued color, which demonstrates that feature-based selection can take place in the absence of spatial attention. To ensure separable color and location processes Andersen et al.^66^ advised that cued target and uncued distractor objects must be presented concurrently and at random locations in the search displays. We followed this advice in the current experiment and presented target and distractors randomly at four out of eight possible stimulus locations in each trial to maximize the spatial variability of the search arrays between trials (while keeping all targets and distractors at an equal eccentricity from fixation to avoid perceptual confounds in the ERPs). We have taken every possible precaution for our experimental design to tap into spatial or feature-based selection exclusively, but it must be noted that our experiment does not completely rule out the possibility that location- and feature-specific effects were integrated in our measures and therefore caused the null effects.

## Conclusions

Taken together, our results suggest that target locations can be pre-activated in working memory to guide attentional selection efficiently to one or two target locations. Importantly, attentional guidance by such location-specific templates appears to be comparable to guidance by feature-specific templates that are designed to guide attention in a spatially global fashion across the visual field. Our data contradict the assumption that spatial selection is superior to feature-based selection but show that this critically depends on controlled parameters of task difficulty. Our findings align with accounts that assume a common neuronal network during preparation for location and color search, but regard spatial and feature-based selection mechanisms as independent. However, further research assessing selection efficiency in feature/location conjunctions is needed to produce more conclusive evidence about the independence of template-guided spatial and feature-based selection mechanisms.

## Supplementary Information

Supplementary Information.

## Data Availability

The datasets used and/or analyzed during the current study available from the corresponding author.
