# Supplementary material for: Attentional templates for target features versus locations
Source: Sci Rep. 2024 Sep 27;14:22306. doi: 10.1038/s41598-024-73656-6 (PMC11437174; doi:10.1038/s41598-024-73656-6)
Supplement: Supplementary file 1 — Supplementary Information. [file 41598_2024_73656_MOESM1_ESM.docx]

**Supplementary materials**

**1. Pilot 1: Exogenous color and location cues**

**1.1. Methods**

*Participants*. Twelve Durham University students took part in this behavioral pilot. Participants gave informed written consent prior to testing. They were aged between 19 and 41 years (*M*=26.5, *SD*=8.5) and two were left-handed. Seven of the participants were female, five were male. All participants had normal or corrected-to-normal visual acuity and normal color vision (tested with the Ishihara color vision test^39^). They either received course credits or were paid (£10 per hour) as a compensation for their time. The experiment was approved by the Ethics Committee of the Psychology Department at Durham University and was conducted in accordance with the Declaration of Helsinki.

*Stimuli and procedure*. Stimuli were presented on a 24-in BenQ LCD monitor (resolution: 1280×1024 pixels; 75Hz refresh rate) at a viewing distance of approximately 100cm. Stimulus presentation, timing and response collection was controlled by a LG Pentium PC running under Windows 7, using MATLAB and the Cogent 2000 toolbox. All stimuli were presented on a black background. A central grey fixation point (CIE x/y color coordinates: .313/.352; 0.3°x0.3° of visual angle) was continuously shown throughout each experimental block. Each trial began with the presentation of a cue display (50ms), which signaled the target color(s) or location(s) in each trial. The cue display was followed by an 950ms retention period, the response-relevant search display (50ms), and a 1800ms inter-trial interval during which responses were collected (Figure S1).


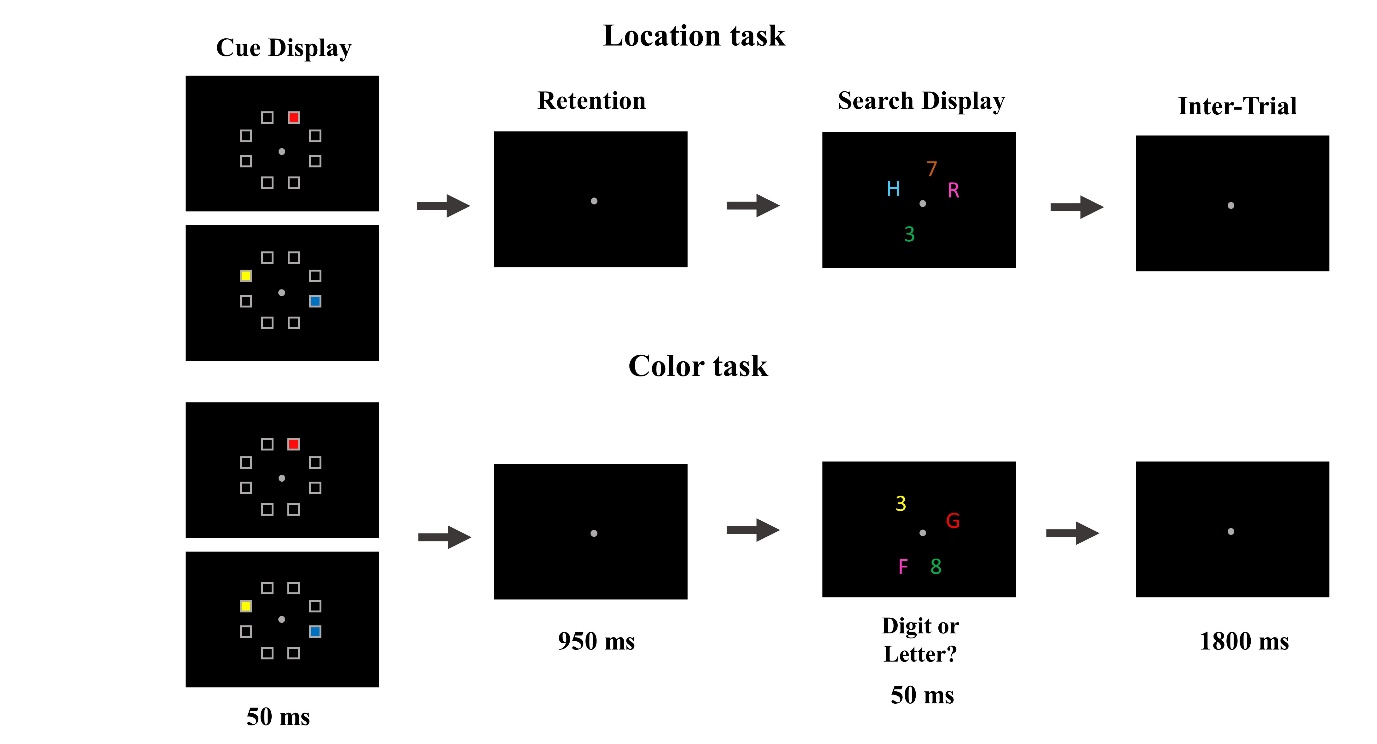


**Figure S1.** Schematic illustration of the time course and the stimuli presented in each trial of the location (top panel) and color task (bottom panel).

Search displays contained four items (0.6°x 0.6° in size), which were located on an imaginary circle (1.3° eccentricity from fixation with respect to the center of the bars) with eight possible equidistant stimulus locations at 22.5°, 67.5°, 112.5°, 157.5°, 202.5°, 247.5°, 292.5°, and 337.5° (to the right from the vertical midline). In each trial, two locations in the left and two in the right hemifield were occupied by two digits and two letters out of eight possible digits (2, 3, 4, 5, 6, 7, 8, 9) and letters (A, K, G, H, N, F, R, Y). Digit and letter identities were randomly chosen in each trial, without replacement. Each search item had a different color. There were six possible target colors: red (CIE x/y color coordinates: .629/.344), green (.283/.613), blue (.175/.202), pink (.306/.194), yellow (.461/.478), or cyan (.214/.340). Grey (.313/.342) and brown (.422/.462) served as additional non-target colors to match the number of available stimulus colors and locations in both tasks (eight in each task). All colors were equiluminant (∼11.0cd/m^2^). Search displays were preceded by cue displays which composed of eight grey square placeholders (0.6°x0.6°) located at the eight possible stimulus locations of the search display. In the *Color Task*, one (low-load) or two (high-load) random placeholders were filled with the relevant color(s) for the upcoming search. In the *Location Task*, one (low-load) or two (high-load) of the placeholders at the relevant target locations for the upcoming search were shown in one or two random colors. To avoid spatial cueing in the color task and color priming in the location task, the target would never appear at any of the cued locations in the color task and never in any of the cued colors in the location task. Even though two colors/locations were cued in the high-load conditions of the color/location task, search displays only ever contained one target that randomly matched the color/location of one of the cues. The other unused cue color/location never appeared in the search display. Participants’ task was to report the identity (digit/letter) of the cued target by pressing the arrow up/down key on a standard keyboard as quickly but accurately as possible. The response-to-key mapping (vertical/horizontal response on arrow up/down key) and the hand-to-key mapping (left/right hand on arrow up/down key) were counterbalanced across participants but was kept constant for each participant for the duration of the whole experiment. The color and location tasks were tested in different blocks and the order of task was counterbalanced across participants. Within each block, all template load (one/two) and target side (left/right hemifield) combinations appeared equally often (12 trials for each combination), resulting in a total of 48 trials per block. Twelve blocks were tested for a total of 576 trials per task. Before each task, participants received a few practice trials until they felt comfortable with the task (~24 trials).

**1.2. Results**

Anticipatory (<200ms) and very slow responses (>1500ms) were removed from analysis (1.6% and 1.2% of all trials in the color and location task, respectively). RTs and error rates were sent to two separate repeated measures ANOVAs with the factors Task (color vs location) and Template Load (low vs high). The ANOVA on the RTs revealed a main effect for Template Load, *F* (1,11) = 259.8, *p* < .001, *η^2^_p_* = .95, indicated that participants were slower in high-load (861ms) compared to low-load trials (749ms). There was also a main effect of Task, *F* (1,11) = 5.2, *p* = .043, *η^2^_p_* = .32, showing that participants responded faster in the location than in the color task (787 vs. 822ms). However, the Task x Load interaction was also significant, *F* (1,11) = 10.0, *p* = .009, *η^2^_p_* = .47, because the RT advantage in the location vs color task was more pronounced in high-load (835 vs 887ms), *t*(1,11) = 3.1, *p* = .010, *d* = 0.89, as compared to low-load trials (739 vs 757ms), *t*(1,11) = 1.2, *p* = .272, *d* = 0.33. The ANOVA on error rates also revealed a main effect of Template Load, *F* (1,11) = 86.9, *p* < .001, *η^2^_p_* = .88, with higher error rates in high- (13.1%) versus low-load trials (4.7%). The main effect for Task was not significant, *F* (1,11) = 2.1, *p* = .177, *η^2^_p_* = .16, but the Task x Template Load reached significance, *F* (1,11) = 27.3, *p* < .001, *η^2^_p_* = .71. As for RTs, the task effect was more pronounced in high-load trials (10.7 vs 15.4% in location vs color trials), *t* (1,11) = 2.7, *p* = .002, *d* = 0.25, than in low-load trials (4.2% vs 5.1% in location vs color trials), *t* (1,11) = -0.9, *p* = .393, *d* = 0.78.

**2. Pilot 2: Endogenous color and location cues**

**2.1. Methods**

All methodological procedures were identical to Pilot 1 with the few exceptions stated below.

*Participants*. Four participants took part in Pilot 2. They were aged between 20 and 35 years (*M*=25.0, *SD*=6.1), all of them were right-handed. One of the participants was female, three were male.

*Stimuli and procedure*. Cue displays in Pilot 2 used central (endogenous) as opposed to peripheral (exogenous) cues both in the color and location tasks. Cue placeholders were now eight grey circles (0.3°x0.3°) that were presented on an imaginary circle much closer to fixation than in Pilot 1 (eccentricity of 0.4° from fixation; see Figure S2).


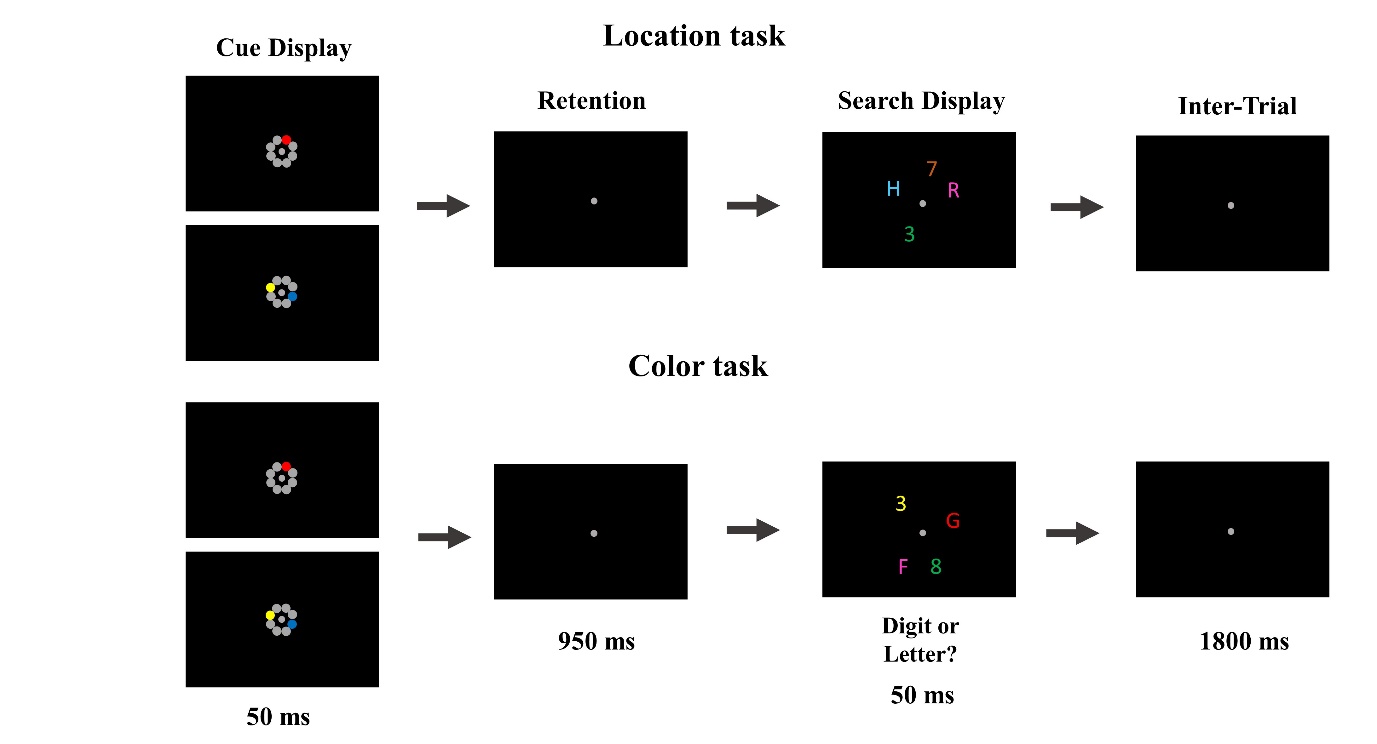


**Figure S2.** Schematic illustration of the time course and the stimuli presented in each trial of the location (top panel) and color task (bottom panel).

**2.2. Results**

Anticipatory (<200ms) and very slow responses (>1500ms) were removed from analysis (1.7% and 0.7% of all trials in the color and location task, respectively). RTs and error rates were sent to two separate repeated measures ANOVA with the factors Task (color vs location) and Template Load (low vs high). The RT ANOVA revealed a main effect of Template Load, *F* (1,3) = 233.1, *p* < .001, *η^2^_p_* = .98, indicating that participants were slower in high-load (825ms) than low-load trials (705ms). There was again a main effect of Task, *F* (1,3) = 16.6, *p* = .002, *η^2^_p_* = .85, showing that participants responded faster in the location than in the color task (796 vs 735ms). The Task x Load interaction was not significant, *F* (1,3) = 0.86, *p* = .420, *η^2^_p_* = .22. The ANOVA on error rates also revealed a main effect of Template Load, *F* (1,3) = 19.3, *p* = .022, *η^2^_p_* = .86, with higher error rates in high-load (14.5%) than low-load trials (5.9%). The main effect for Task was not significant, *F* (1,3) = 4.3, *p* = .129, *η^2^_p_* = .59, but the Task x Template Load reached significance, *F* (1,3) = 31.1, *p* = .011, *η^2^_p_* = .91. Error rates were higher in the color vs location task in both high-load (16.8 vs 12.1%) and low-load trials (6.2 vs 5.5%), but not reliably, both *t*(1,3)<3.03, *p*> .056, *d*< 1.5.
